# Supplementary figures and images for: Using Whole-Genome Sequence Data to Predict Quantitative Trait Phenotypes in Drosophila melanogaster
Source: PLoS Genet. 2012 May 3;8(5):e1002685. doi: 10.1371/journal.pgen.1002685 (PMC3342952; doi:10.1371/journal.pgen.1002685)

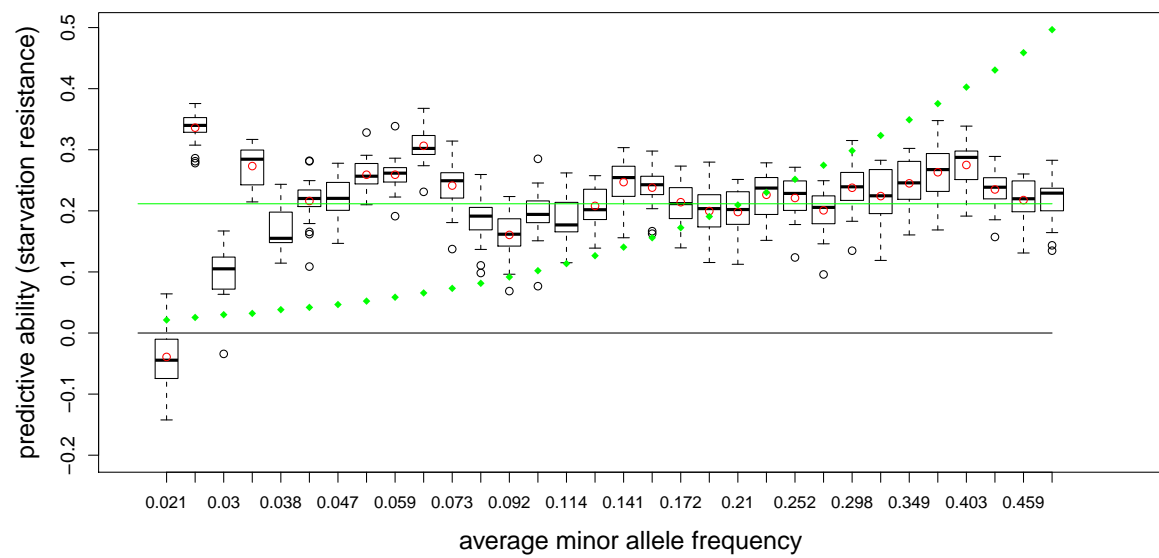

Supplement: Figure S1 — Predictive ability of 5-fold CV with GBLUP for starvation resistance using different set of SNPs with different average minor allele frequencies. Each boxplot shows the average predictive abilities for replicates of 5-fold CV using GBLUP and SNPs with different average minor allele frequencies. The different average minor allele frequencies are plotted as green dots. To choose the SNPs for each bin of minor allele frequency the SNPs were sorted by minor allele frequency and then divided into blocks, i.e. each bin contained SNPs. The horizontal green line indicates the average accuracy obtained using every SNP (resulting in SNPs as well), which was . (PDF) [file pgen.1002685.s001.pdf]

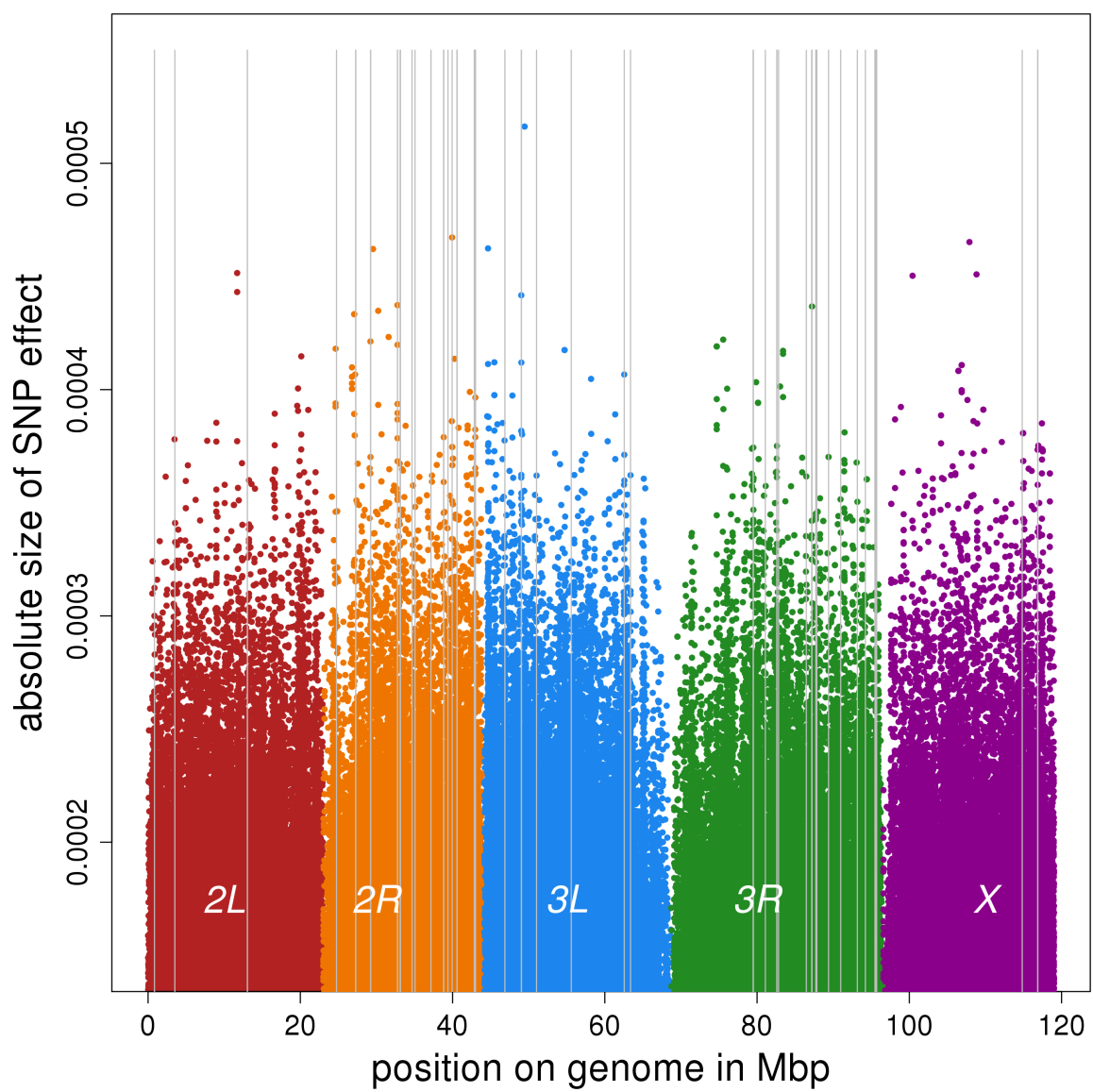

Supplement: Figure S2 — Manhattan plot of the estimated SNP effects for starvation resistance for different chromosomes. The SNP effects were estimated using the GBLUP approach and sex-averaged phenotypic values of lines. Vertical lines indicate the significant SNP positions according to the GWAS of [27] using sex-pooled records. (PDF) [file pgen.1002685.s002.pdf]

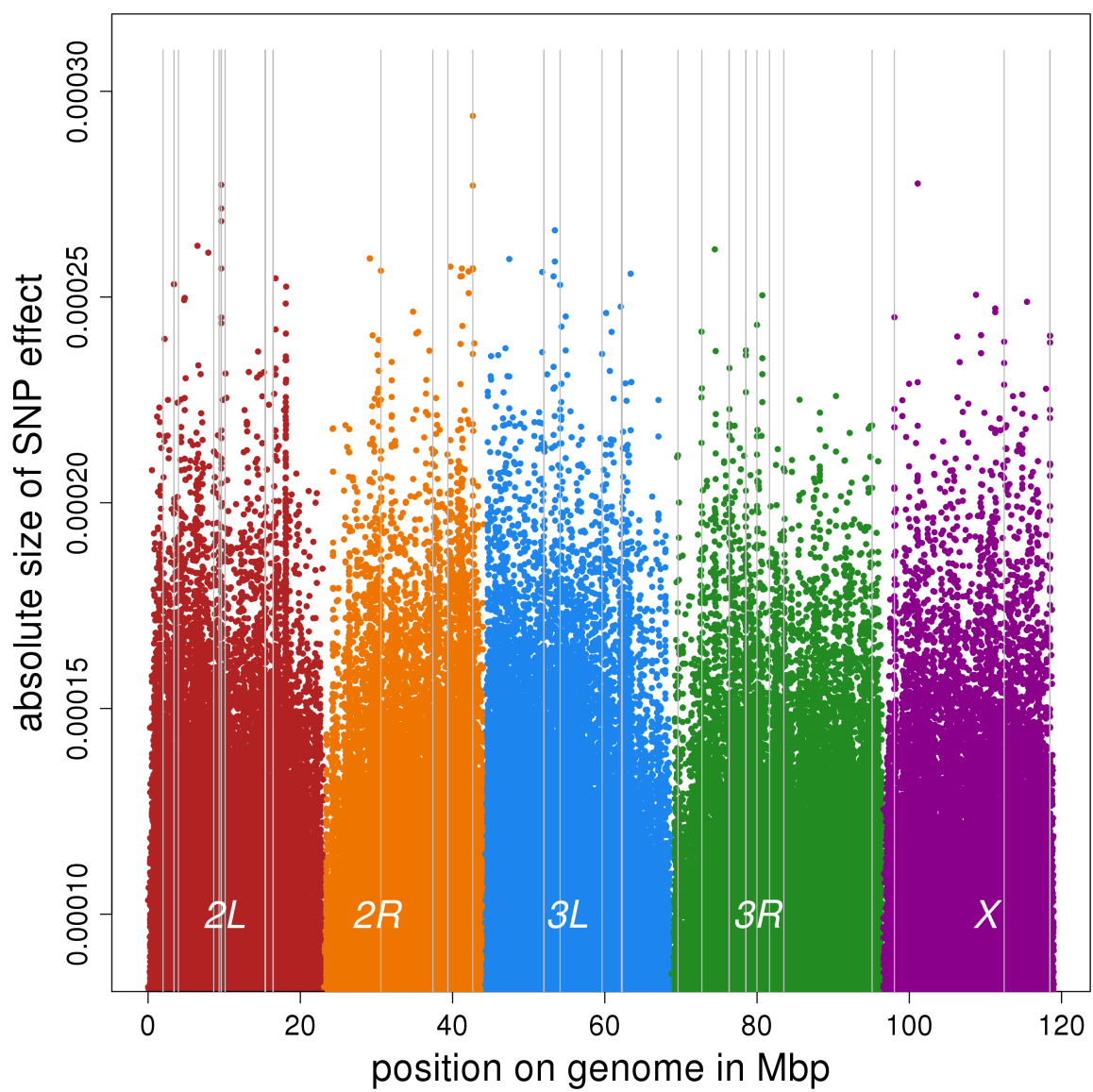

Supplement: Figure S3 — Manhattan plot of the estimated SNP effects for startle response for different chromosomes. The SNP effects were estimated using the GBLUP approach and sex-averaged phenotypic values of lines. Vertical lines indicate the significant SNP positions according to the GWAS of [27] using sex-pooled records. (PDF) [file pgen.1002685.s003.pdf]
