# Supplementary material for: Using Whole-Genome Sequence Data to Predict Quantitative Trait Phenotypes in Drosophila melanogaster
Source: PLoS Genet. 2012 May 3;8(5):e1002685. doi: 10.1371/journal.pgen.1002685 (PMC3342952; doi:10.1371/journal.pgen.1002685)
Supplement: Table S1 — Mean and standard deviation of phenotypic values and of the number of individual records per line. (PDF) [file pgen.1002685.s004.pdf]

Table S1. Mean and standard deviation of phenotypic values and of the number of individual records per line.

	starvation resistance		startle response	
	phen. value[1]	# rec. per line[2]	phen. value	# rec. per line
all	52.5 ± 10.7	104.1 ± 21.5	29.4 ± 6.6	80.1 ± 7.4
female	44.9 ± 10.0	52.2 ± 11.2	29.2 ± 6.7	40.2 ± 3.9
male	60.2 ± 13.4	51.8 ± 10.8	29.5 ± 6.7	39.8 ± 4.3

[1] Phenotypic values were calculated as the average of the medians of male and female records ("all") or as medians of female or male records separately.

[2] Number of records per line.
