# Supplementary material for: Using Whole-Genome Sequence Data to Predict Quantitative Trait Phenotypes in Drosophila melanogaster
Source: PLoS Genet. 2012 May 3;8(5):e1002685. doi: 10.1371/journal.pgen.1002685 (PMC3342952; doi:10.1371/journal.pgen.1002685)
Supplement: Table S2 — Variance components and heritabilities estimated from GBLUP using all lines. Variance components were estimated by maximum likelihood using the R-package “RandomFields” and its function “fitvario.” (PDF) [file pgen.1002685.s005.pdf]

Table S2. Variance components and heritabilities estimated from GBLUP using all lines. Variance components were estimated by maximum likelihood using the R-package "RandomFields" and its function "fitvario".

	starvation resistance			startle response		
	$\hat{\sigma}_g^2$	$\hat{\sigma}_e^2$	$\hat{h}^2_{\text{GBLUP}}$	$\hat{\sigma}_g^2$	$\hat{\sigma}_e^2$	$\hat{h}^2_{\text{GBLUP}}$
all	62.6	0	1	21.7	0	1
female	91.2	0	1	22.4	0	1
male	57.9	0	1	22.5	0	1

Variance components were estimated using the average of the medians of male and female records ("all") or the medians of female or male records separately as phenotypic data.
