# Supplementary material for: Using Whole-Genome Sequence Data to Predict Quantitative Trait Phenotypes in Drosophila melanogaster
Source: PLoS Genet. 2012 May 3;8(5):e1002685. doi: 10.1371/journal.pgen.1002685 (PMC3342952; doi:10.1371/journal.pgen.1002685)
Supplement: Table S3 — Results of variance component estimation using ASReml for starvation resistance. Different linear models for individual trait records were investigated. (PDF) [file pgen.1002685.s006.pdf]

Table S3. Results of variance component estimation using ASReml for starvation resistance. Different linear models for individual trait records were investigated.

Starvation Resistance	$\hat{\sigma}^2_{\text{line}}$	$\hat{\sigma}^2_{\text{sex*line}}$	$\hat{\sigma}^{2,1}_{\text{rep(sex*line)}}$	$\hat{\sigma}^2_g$	$\hat{\sigma}^2_{g\times g}$	$\hat{\sigma}^2_{\text{residual}}$	$\ln(L)^2$	$\hat{H}^{2,3}_{\text{Model 1}}$	$\hat{h}^{2,4}_{\text{Model 2/3}}$
Model 1	87.97[5] (12.74)	39.65 (5.06)	17.00 (1.01)			88.00 (1.03)	-46097.51	0.59 (0.02)	
Model 2	0.00	39.35 (4.99)	17.00 (1.01)	43.13 (6.32)		88.00 (1.03)	-46093.63**,[6]		0.25 (0.03)
Model 3	0.00	39.35 (4.98)	17.00 (1.01)	43.13 (6.32)	0.00	88.00 (1.03)	-46093.63		0.25 (0.03)
Model 1 f[7]	167.62 (19.63)		18.22 (1.64)			113.92 (1.88)	-24174.10	0.60 (0.03)	
Model 2 f	9.07 (28.35)		18.22 (1.64)	78.24 (18.02)		113.92 (1.88)	-24170.31**		0.39 (0.11)
Model 3 f	9.08 (28.34)		18.22 (1.64)	78.24 (18.02)	0.00	113.92 (1.88)	-24170.31		0.39 (0.11)
Model 1 m[8]	87.57 (10.40)		15.73 (1.21)			61.89 (1.02)	-21617.19	0.59 (0.03)	
Model 2 m	5.23 (15.49)		15.73 (1.21)	40.51 (9.68)		61.89 (1.02)	-21613.34**		0.38 (0.11)
Model 3 m	5.23 (15.49)		15.73 (1.21)	40.51 (9.68)	0.00	61.89 (1.02)	-21613.34		0.38 (0.11)

[1] or “rep(line)” if factor sex is not included

[2] loglikelihood

[3] broad-sense heritability, standard errors in parentheses

[4] narrow-sense heritability, standard errors in parentheses

[5] estimated variance components, standard errors in parentheses

[6] The superscript ** indicates the 1%-significance of Model 2 compared to the Model 1 without g-component based on a likelihood ratio test.

[7] Only measurements of female Drosophila were used.

[8] Only measurements of male Drosophila were used.
