# Supplementary material for: Using Whole-Genome Sequence Data to Predict Quantitative Trait Phenotypes in Drosophila melanogaster
Source: PLoS Genet. 2012 May 3;8(5):e1002685. doi: 10.1371/journal.pgen.1002685 (PMC3342952; doi:10.1371/journal.pgen.1002685)
Supplement: Table S4 — Results of variance component estimation using ASReml for startle response. Different linear models for individual trait records were investigated. (PDF) [file pgen.1002685.s007.pdf]

**Table S4.** Results of variance component estimation using ASReml for startle response. Different linear models for individual trait records were investigated.

| Startle Response | $\hat{\sigma}^2_{\text{line}}$ | $\hat{\sigma}^2_{\text{sex}*\text{line}}$ | $\hat{\sigma}^{2,1}_{\text{rep(sex}*\text{line)}}$ | $\hat{\sigma}^2_g$ | $\hat{\sigma}^2_{g\times g}$ | $\hat{\sigma}^2_{\text{residual}}$ | $\ln(L)^2$ | $\hat{H}^{2,3}_{\text{Model 1}}$ | $\hat{h}^{2,4}_{\text{Model 2/3}}$ |
|---|---|---|---|---|---|---|---|---|---|
| Model 1 | 33.49[5] (4.38) | 0.00 | 17.81 (1.26) | | | 25.70 (0.33) | -27320.93 | 0.57 (0.03) | |
| Model 2 | 0.00 | 0.00 | 17.79 (1.26) | 16.45 (2.17) | | 25.70 (0.33) | -27317.45**,[6] | | 0.39 (0.03) |
| Model 3 | 0.00 | 0.00 | 17.78 (1.26) | 12.98 (11.47) | 1.68 (5.52) | 25.70 (0.33) | -27317.41 | | 0.32 (0.24) |
| Model 1 f[7] | 24.07 (4.72) | | 27.96 (3.33) | | | 25.26 (0.46) | -13746.53 | 0.49 (0.05) | |
| Model 2 f | 0.00 | | 27.61 (3.25) | 11.89 (2.34) | | 25.26 (0.46) | -13743.71* | | 0.32 (0.04) |
| Model 3 f | 0.00 | | 27.61 (3.25) | 11.89 (2.34) | 0.00 | 25.26 (0.46) | -13743.71 | | 0.32 (0.04) |
| Model 1 m[8] | 27.26 (4.74) | | 23.23 (2.81) | | | 26.13 (0.48) | -13682.01 | 0.51 (0.04) | |
| Model 2 m | 0.00 | | 23.03 (2.77) | 13.34 (2.35) | | 26.13 (0.48) | -13678.87* | | 0.34 (0.04) |
| Model 3 m | 0.00 | | 23.03 (2.77) | 13.34 (2.35) | 0.00 | 26.13 (0.48) | -13678.87 | | 0.34 (0.04) |

[1] or "rep(line)" if factor sex is not included

[2] loglikelihood

[3] broad-sense heritability, standard errors in parentheses

[4] narrow-sense heritability, standard errors in parentheses

[5] estimated variance components, standard errors in parentheses

[6] The superscripts $*$ and $**$ indicate the 5%- and 1%-significance of Model 2 compared to the Model 1 without $g$-component based on a likelihood ratio test.

[7] Only measurements of female Drosophila were used.

[8] Only measurements of male Drosophila were used.
