# Supplementary material for: Using Whole-Genome Sequence Data to Predict Quantitative Trait Phenotypes in Drosophila melanogaster
Source: PLoS Genet. 2012 May 3;8(5):e1002685. doi: 10.1371/journal.pgen.1002685 (PMC3342952; doi:10.1371/journal.pgen.1002685)
Supplement: Text S1 — We give more details on the formula of [52] for the expected linkage disequilibrium as well as the derivation of the number of independently segregating chromosome segments [9] and the expected accuracy of prediction [20] in the case of D. melanogaster. We also derive the expected value of the genomic relationship matrix of [8] and show that , where is the numerator relationship matrix. (PDF) [file pgen.1002685.s008.pdf]

Text S1

Supporting text to

Using Whole Genome Sequence Data to Predict Quantitative Trait Phenotypes in *Drosophila melanogaster*

Ulrike Ober[1,∗], Julien F. Ayroles[2,3], Eric A. Stone[2], Stephen Richards[4], Dianhui Zhu[4], Richard A. Gibbs[4], Christian Stricker[5], Daniel Gianola[6], Martin Schlather[7], Trudy F. C. Mackay[2] and Henner Simianer[1]

1 Animal Breeding and Genetics Group, Georg-August-University Göttingen, 37075 Göttingen, Germany

2 Department of Genetics, North Carolina State University, Raleigh, NC 27695-7614, United States of America

3 Department of Organismic and Evolutionary Biology, Harvard University, Cambridge, MA 02138, United States of America

4 Human Genome Sequencing Center, Baylor College of Medicine, Houston, TX 77006, United States of America

5 agn Genetics GmbH, Davos 7260, Switzerland

6 Department of Animal Sciences, University of Wisconsin-Madison, WI 53706, United States of America

7 Institute for Mathematics, University of Mannheim, 68131 Mannheim, Germany

∗ E-mail: uober@math.uni-goettingen.de

Text S1

When working with *D. melanogaster*, we have to pay attention to a specific characteristic: Male individuals do not recombine, *i.e.* the overall recombination rate c equals $\frac{1}{2}c_f$, where c_f is the recombination rate in female individuals. Moreover, the genome length in Morgans is $L = 0.5L_f$, where L_f is the length of the female genome in Morgans.

The formula of [1] for the expected linkage disequilibrium:

The following formula for the expected LD in a population based on the effective population size N_e was proposed by [1]:

$$\mathbb{E}(r^2) \approx \frac{1}{1 + 4N_e c} \quad \Leftrightarrow \quad N_e = \frac{\frac{1}{\mathbb{E}(r^2)} - 1}{4c} \tag{1}$$

Here, N_e corresponds to an effective population size $t = \frac{1}{2c}$ generations ago [2]. Using $c = \frac{1}{2}c_f$ we obtain

$$\mathbb{E}(r^2) = \frac{1}{1 + 2N_e c_f} \quad \Leftrightarrow \quad N_e = \frac{\frac{1}{\mathbb{E}(r^2)} - 1}{2c_f},$$

$t = \frac{1}{c_f}$ generations ago.

If this formula is used to estimate N_e based on a finite sample of individuals, one should adjust for the chromosome sample size [3], which equals the number of individuals n in the case of inbred lines. Then,

$$\mathbb{E}(r^2) = \frac{1}{1 + 2N_e c_f} + \frac{1}{n} \quad \Leftrightarrow \quad N_e = \frac{\frac{1}{\mathbb{E}(r^2) - \frac{1}{n}} - 1}{2c_f}.$$

Note that when applying this formula to the DGRP population, the estimated N_e is not the effective population size of the local wild population the actual lines were sampled from, but the effective population size of an idealized population having the same structure of LD as the DGRP inbred lines. This means that we consider the 157 independent gametes of the DGRP inbred lines as a random sample of this idealized population.

Several derivations of the above formula have been suggested in the last forty years [1,4–7] and simulation studies have shown that the simulated values of r^2 agree reasonably well with the expectations based on this formula. However, we found that all derivations mentioned above have serious shortcomings from a mathematical point of view. Similar concerns over the exact validity of the formula and their derivations

were recently raised by [6], p. 185, cf. also the manuscript published on John Sved's personal homepage (`http://www.handsongenetics.com/PIFFLE/LinkageDisequilibrium.pdf`). We clearly think that further research is needed to find a substantiated proof and that results based on this formula should therefore be taken with caution.

Derivation of the number of independently segregating chromosome segments M_e and the expected accuracy of prediction $\mathbb{E}(\rho)$: The formula [8] for the expected accuracy of genomic prediction $\mathbb{E}(\rho)$ with GBLUP depends on the number of independently segregating genome segments M_e [9]:

$$\mathbb{E}(\rho) = \sqrt{\frac{N_p h^2}{N_p h^2 + M_e}}$$

We will derive how M_e can be calculated in the case of *D. melanogaster*. The general derivation of M_e for a diploid population is given in [9] and based on the Sved-formula [1]. Central in the derivation is the calculation of the double integral over the formula for $\mathbb{E}(r^2)$. In general, one can verify that

$$\frac{1}{a_1^2}\int_0^{a_1}\int_0^{a_1} \frac{1}{a_3 + a_2|x_1 - x_2|} dx_1 dx_2 = \frac{2(a_3 + a_1 a_2)\ln(a_3 + a_1 a_2)}{a_1^2 a_2^2} - \frac{2a_3 \ln(a_3)}{a_1^2 a_2^2} - \frac{2\ln(a_3)}{a_1 a_2} - \frac{2}{a_1 a_2},$$

for arbitrary constants a_1, a_2, a_3 with $a_1, a_2 > 0$. If $a_3 \in \{1, 2\}$ and if a_2 is large enough, the double integral is approximately

$$\frac{1}{a_1^2}\int_0^{a_1}\int_0^{a_1} \frac{1}{a_3 + a_2|x_1 - x_2|} dx_1 dx_2 \approx \frac{2(a_1 a_2)\ln(a_1 a_2)}{a_1^2 a_2^2} = \frac{2\ln(a_1 a_2)}{a_1 a_2}.$$

Following the derivation of [9], we need to calculate the double integral over eq. (1) and displace c by the distance $|x_1 - x_2|$ which leads to

$$\frac{1}{L^2}\int_0^{L}\int_0^{L} \frac{1}{1 + 4N_e|x_1 - x_2|} dx_1 dx_2 = \frac{1}{L_f^2}\int_0^{L_f}\int_0^{L_f} \frac{1}{1 + 2N_e|x_1 - x_2|} dx_1 dx_2 \approx \frac{2\ln(L_f 2N_e)}{L_f 2N_e} = \frac{\ln(L_f 2N_e)}{L_f N_e}.$$

Here, the first equality holds because of the transformation formula and the identity $L = \frac{1}{2}L_f$ in the case of *D. melanogaster*. Using this result, M_e can be derived as in [9], leading to

$$M_e = \frac{N_e L_f}{\ln(2N_e L_f)}.$$

Hence, the formula of [8] for the expected accuracy of prediction in the case of *D. melanogaster* equals

$$\mathbb{E}(\rho) = \sqrt{\frac{N_p h^2}{N_p h^2 + M_e}} = \sqrt{\frac{N_p h^2}{N_p h^2 + \frac{N_e L_f}{\ln(2N_e L_f)}}},$$

where N_p is the size of the training set and h^2 is the narrow-sense heritability of the trait estimated from the GBLUP model.

The expected value of the genomic relationship matrix of [10]: In this section we will show that the expected value of the genomic relationship matrix $\mathbf{G}$ of [10] is given by the additive relationship matrix $\mathbf{A}$, *i.e.*

$$\mathbb{E}(\mathbf{G}) = \mathbf{A}.$$

Following [10], $\mathbf{G}$ is defined as

$$\mathbf{G} = \frac{(\mathbf{M}-\mathbf{P})(\mathbf{M}-\mathbf{P})^T}{2\sum_{k=1}^{s} p_k(1-p_k)},$$

where $\mathbf{M}$ is the $(n \times s)$-matrix of SNP genotype vectors for the n lines with the s SNPs coded as $-1, 1$ and the kth column of $\mathbf{P}$ is $(2(p_k - 0.5), \ldots, 2(p_k - 0.5))^T$, where p_k is the frequency of the second allele at locus k for $k = 1, \ldots, s$.

Let $\mathbf{m}_i$ be the vector of SNP genotypes of individual i, *i.e.* $\mathbf{m}_i = (m_{i1}, \ldots, m_{is})$. Then, $\mathbf{M} = (\mathbf{m}_1, \ldots, \mathbf{m}_n)^T$. We consider the case of fully homozygous individuals due to full sib mating. Then, the genotype m_{ik} of individual $i = 1, \ldots, n$ at locus $k = 1, \ldots, s$ can be considered as a discrete random variable with values $-1, 1$ and probabilities $(1 - p_k), p_k$, and it is

$$\mathbb{E}(m_{ik}) = -(1 - p_k) + p_k = 2p_k - 1$$

for all $i = 1, \ldots, n$. Moreover, we have

$$\sum_{k=1}^{s} \text{Cov}(m_{ik}, m_{jk}) = a_{ij} \sum_{k=1}^{s} \sigma^2_{\mathbf{m}_{\bullet k}},$$

where a_{ij} is the coefficient of relationship between individuals i and j, and $\sigma^2_{\mathbf{m}_{\bullet k}}$ is the variance of the

genotype variable $m_{\bullet k}$ at locus k of the original base-population, see [11] for a derivation of the covariance between relatives under full sib mating.

The variance of $m_{\bullet k}$ in the base population is equal to the variance of a random variable with values $-1, 0, 1$ and probabilities $(1-p_k)^2, 2p_k(1-p_k), p_k^2$, which equals

$$\begin{aligned}
\sigma^2_{\mathbf{m}_{\bullet k}} &= \mathbb{E}(\mathbf{m}^2_{\bullet k}) - \mathbb{E}(\mathbf{m}_{\bullet k})^2 \\
&= (-1)^2 \cdot (1-p_k)^2 + 0^2 \cdot 2p_k(1-p_k) + 1^2 \cdot p_k^2 - \left(-1 \cdot (1-p_k)^2 + 0 \cdot 2p_k(1-p_k) + 1 \cdot p_k^2\right)^2 \\
&= 2p_k(1-p_k).
\end{aligned}$$

This leads to

$$\sum_{k=1}^{s} \mathrm{Cov}(m_{ik}, m_{jk}) = a_{ij} \sum_{k=1}^{s} 2p_k(1-p_k). \tag{2}$$

Define $D := 2\sum_{k=1}^{s} p_k(1-p_k)$. The expected value of $\mathbf{G}$ can now be calculated as

$$\begin{aligned}
[\mathbb{E}(\mathbf{G})]_{ij} &= \left[\mathbb{E}\left(\frac{(\mathbf{M}-\mathbf{P})(\mathbf{M}-\mathbf{P})^T}{D}\right)\right]_{ij} \\
&= \frac{1}{D}\mathbb{E}\left[(\mathbf{m}_i - (2(p_1-0.5), \dots, 2(p_s-0.5))) \cdot (\mathbf{m}_j - (2(p_1-0.5), \dots, 2(p_s-0.5)))^T\right] \\
&= \frac{1}{D}\mathbb{E}\left[((m_{i1}, \dots, m_{is}) - \mathbb{E}(m_{i1}, \dots, m_{is})) \cdot ((m_{j1}, \dots, m_{js}) - \mathbb{E}(m_{j1}, \dots, m_{js}))^T\right] \\
&= \frac{1}{D}\sum_{k=1}^{s} \mathbb{E}\left[(m_{ik} - \mathbb{E}(m_{ik})) \cdot (m_{jk} - \mathbb{E}(m_{jk}))\right] \\
&= \frac{1}{D}\sum_{k=1}^{s} \mathrm{Cov}(m_{ik}, m_{jk}) \\
&= \frac{1}{2\sum_{k=1}^{s} p_k(1-p_k)} \left(a_{ij}\sum_{k=1}^{s} 2p_k(1-p_k)\right), \text{using eq. (2)} \\
&= a_{ij}
\end{aligned}$$

for $i, j = 1, \dots, n$, *i.e.* $\mathbb{E}(\mathbf{G}) = \mathbf{A}$.

The derivation presented above was for the case of fully homozygous individuals due to full sib mating. The identity $\mathbb{E}(\mathbf{G}) = \mathbf{A}$ can analogously be derived for a non-homozygous population. Then, the genotype m_{ik} of individual i at locus k can be considered as a discrete random variable with values $-1, 0, 1$ and

probabilities $(1-p)^2, 2p_k(1-p_k), p_k^2$.

References

1. Sved JA (1971) Linkage disequilibrium and homozygosity of chromosome segments in finite populations. Theor Popul Biol 2: 125-141.

2. Hayes BJ, Visscher PM, McPartland HC, Goddard ME (2003) Novel multilocus measure of linkage disequilibrium to estimate past effective size. Genome Res 13: 635–643.

3. Hill WG, Weir BS (1980) Effect of mating structure on variation in linkage disequilibrium. Genetics 95: 477-488.

4. Sved JA, Feldmann MW (1973) Correlation and probability methods for one and two loci. Theor Popul Biol 4: 129-132.

5. Tenesa A, Navarro P, Hayes BJ (2007) Recent human effective population size estimated from linkage disequilibrium. Genom Res 17: 520–526.

6. Sved JA (2008) Linkage disequilibrium and its expectation in human populations. Twin Research and Human Genetics 12: 35-43.

7. Sved JA (2009) Correlation measures for linkage disequilibrium within and between populations. Genet Res : 183-192doi:10.1017/S0016672309000157.

8. Daetwyler HD, Pong-Wong R, Villanueva B, Woolliams JA (2010) The impact of genetic architecture on genome-wide evaluation methods. Genetics 185: 1021–1031.

9. Goddard M (2009) Genomic selection: Prediction of accuracy and maximisation of long-term response. Genetica 185: 1021–1031.

10. VanRaden PM (2008) Efficient methods to compute genomic predictions. J Dairy Sci 91: 4414-4423.

11. Cornelius PL, Dudley JW (1975) Theory of inbreeding and covariances between relatives under fullsib-mating in diploids. Biometrics 31: 169-187.
